# Entropy, Periodicity and the Probability of Primality

**DOI:** 10.3390/e27121204

**Published:** 2025-11-27

**Authors:** Grenville J. Croll

**Affiliations:** Alternative Natural Philosophy Association, Bury St Edmunds IP30 9QX, UK; grenvillecroll@gmail.com

**Keywords:** probability of primality, binary derivative, periodicity, prime number distribution, BiEntropy, randomness and complexity, Riemann Hypothesis

## Abstract

The distribution of prime numbers has long been viewed as a balance between order and randomness. In this work, we investigate the relationship between entropy, periodicity, and primality through the computational framework of the binary derivative. We prove that periodic numbers are composite in all bases except for a single trivial case and establish a set of twelve theorems governing the behavior of primes and composites in terms of binary periodicity. Building upon these results, we introduce a novel scale-invariant entropic measure of primality, denoted p(*s*′), which provides an exact and unconditional entropic probability of primality derived solely from the periodic structure of a binary number and its binary derivatives. We show that p(*s*′) is quadratic, statistically well-defined, and strongly correlated with our earlier BiEntropy measure of binary disorder. Empirical analyses across several numerical ranges demonstrate that the variance in prime density relative to quadratic expectation is small, binormal, and constrained by the central limit theorem. These findings reveal a deep connection between entropy and the randomness of the primes, offering new insights into the entropic structure of number theory, with implications for the Riemann Hypothesis, special classes of primes, and computational applications in cryptography.

## 1. Introduction

Entropy provides a fundamental framework for quantifying order, disorder, and randomness across natural and computational systems. Since Shannon’s original formulation [1], entropy has been widely applied to measure the uncertainty and information content of symbolic sequences, with extensions into fields as diverse as physics, cryptography, biology, and algorithmic complexity.

Recent research has increasingly explored connections between entropy and prime distribution. Kontoyiannis [2] demonstrated that elementary information-theoretic arguments can provide new proofs for classical results in number theory, including Chebyshev’s theorem on prime density, by quantifying how many bits of information about an integer we learn from each of its prime factors. Billingsley’s [3] influential 1973 work established probabilistic connections between prime numbers and Brownian motion, introducing entropy-based methods to number theory, an approach that earned the Lester R. Ford Award for mathematical exposition. Wolf [4] and Szpiro [5] investigated fractal and statistical properties of prime gaps, revealing 1/f noise patterns and hidden structure in prime distributions through entropy-related analysis. More recently, quantum information approaches have identified prime numbers through singular behaviors in linear entanglement entropy [6]. These studies collectively suggest that entropic approaches provide valuable complementary insights into the structure and apparent randomness of prime distributions alongside traditional analytic methods.

In this paper, we explore how basic entropic principles can further illuminate one of mathematics’ most elusive structures—the distribution of prime numbers.

The apparent irregularity of the primes has long invited probabilistic and statistical interpretation. Classical approaches, such as the Prime Number Theorem and the Riemann Hypothesis, express the distribution of primes through asymptotic density and analytic continuation, yet provide little insight into the local structure and apparent randomness of the prime sequence. Recent research has attempted to capture this complexity through entropy-based measures of randomness and symbolic dynamics. Our study contributes to this growing perspective by developing a binary entropic framework that connects periodicity, entropy, and primality.

In earlier work, we introduced the BiEntropy function as a measure of order and disorder in finite binary strings [7]. This measure, later extended through the TriEntropy framework [8], has been applied to diverse domains including knot theory [9], cryptography [10], cellular automata [11], computational neuroscience [12,13] and surface science [14]. Here, we build upon these foundations to develop an entropic probability of primality, p(*s*′), derived from the binary derivative—a discrete operator that transforms a binary sequence into its pairwise XOR-based derivatives.

The binary derivative possesses an important property: it preserves statistical independence and thus acts as an entropy-conserving transformation. We exploit this property to define a set of theorems relating periodicity and compositeness in binary numbers. Specifically, we show that numbers exhibiting periodic structure in their binary representation are necessarily composite, and that primes, in contrast, are asymptotically equidistributed across the binary derivative space. From these results, we derive p(*s*′), a quadratic, scale-invariant function that quantifies the entropic likelihood that a number is prime.

Empirical investigations of p(*s*′) over several domains (up to 2^32^) reveal a close correspondence between predicted and actual prime frequencies, with correlation coefficients exceeding 0.95. The distribution of residuals in entropic prime density is binormal and consistent with the central limit theorem, suggesting that the primes exhibit bounded stochastic variation rather than true randomness. These findings provide a new perspective on the statistical and entropic foundations of primality and open pathways toward entropy-based number classification and cryptographic modeling.

The remainder of this paper is organized as follows. Section 2 defines periodicity in natural numbers and establishes a set of related compositeness theorems. Section 3 introduces the binary derivative and explores its entropic properties. Section 4 defines the entropic probability of primality, p(*s*′), and relates it to the Prime Number Theorem. Section 5 presents empirical analyses across multiple numerical ranges. Section 6 discusses broader implications for entropy, randomness, and the Riemann Hypothesis.

## 2. Periodicity and Compositeness in Binary Numbers

### 2.1. Definition of Periodic Numbers

Consider the numeric string *s* = *s*_1_*s*_2_…*s_n_*. where *s* ∈ N_0_. Let *s* = *ab*, where *a* and *b* are similar numeric strings of equal length *n* ≥ 1. The length of *s* is |*s*| = *n*. The concatenation *s* = *ab* is said to be periodic when *a* = *b*. Periodic numbers represent elementary cases of iterants [15,16], reflecting internal repetition within a numeric structure.

### 2.2. Periodic Numbers Are Composite

**Theorem** **1.** *Periodic numbers are composite in all bases, except for a single trivial case*.

**Proof**. Let *k* = *ab* in base *m*, with *a* = *b* and *a* > 1. Then:

*k* = (*m^n^*⋅*a*) + *b* (1)

Since *a* = *b*, we obtain

*k* = (*m^n^* + 1)⋅*a* (2)

and because *a* > 1, *k* must be composite.

The exception occurs when *a* = *b* = 1, yielding numbers of the form 11, 0101, 001001, in any base, i.e., k = *m^n^* + 1. Every natural number can be represented in this way. □

Hence, periodic numbers are composite in all bases, except for one specific form. In what follows, we restrict our analysis to the binary base *m* = 2, which is particularly well-suited for the analysis of entropy.

### 2.3. n-Periodic Binary Numbers Are Composite

**Theorem** **2.** *Binary numbers of the form 00111100 or 10010110, where the second half is the one’s complement of the first half, also exhibit compositeness.*

*Let*

*b* = *a*′ = 2*^n^* − a − 1. (3)

*Then all numbers of the form s = ab are composite*.

**Proof**. *k* = (2*^n^*⋅*a*) + *b* = (2*^n^*⋅*a*) + (2*^n^* − *a* − 1) (4)

Simplifying gives:

*k* = (*a + 1*)⋅(2*^n^* − 1) (5)

If *a* ≥ 1, *k* is composite.

If *a* = 0, *b* is a binary string of all ones. When *n* is odd, such numbers may correspond to Mersenne primes; otherwise, they remain composite. □

### 2.4. z-Periodic Numbers Are Composite

**Theorem** **3.** *Numbers of the form zab, where a = b and z is an even number of leading zeros, are also composite.*

**Proof**. Removing the leading zeros reduces the number to the periodic form described in Theorem 1. □

### 2.5. Periodic Numbers Are Composite by Construction

Empirical enumeration of all 8-bit binary numbers (reproduced in the Supplementary Materials) confirms that approximately 20% of them exhibit periodicity as defined above and are therefore composite by construction. We ignore the even numbers (*s* = *abz*) and note certain previously observed periodic special cases [17].

### 2.6. Periodicity and Shannon Entropy

The Shannon entropy of a binary sequence *s* = *s*_1_*s*_2_…*s_n_*, where P(*s_i_* = 1) = *p*.

*H*(*p*) = −*p* log_2_ *p* − (1 − *p*) log_2_(1 − *p*) (6)

The Shannon entropy is zero when all the bits are identical (i.e., it is periodic with a period of one) and maximal when *p* = 0.5, corresponding to maximum variety.

Thus, the absence of periodicity corresponds to greater entropy, linking compositeness and entropy in a direct way.

## 3. The Binary Derivative and Its Entropic Properties

### 3.1. Definition of the Binary Derivative

Given a binary string, the first binary derivative, *d*_1_(*s*), is defined as the XOR of the adjacent bits:

*d*_1_(*s*) = *s*_i_ ⊕ *s_i_*_+1_, 1 ≤ *i* < *n* (7)

The *k*-th binary derivative *d_k_*(*s*) is here defined iteratively:

*d_k_*(*s*) = *d*_*k*−1_(*s*), 1 ≤ *k* ≤ *n* − 1 (8)

The sequence {*d*_0_(*s*), *d*_1_(*s*), …, *d*_*n*−1_(*s*)} forms a binary derivative hierarchy D(s), where *d*_0_(*s*) = *s* and *d*_*n*−1_(*s*) is a single bit.

The binary derivative behaves as a finite-difference operator under XOR addition, making it a natural discrete analog of continuous differentiation.

### 3.2. Periodicity and the Zero Derivative

**Theorem** **4.** *A binary number is periodic with period 2^n^ if and only if one of its derivatives, d_k_(s), equals zero for some 0 ≤ k < 2^n^.*

**Proof**. The proof exactly follows Nathanson’s Lemma 6 [18]. □

This connects periodicity to a total loss of informational entropy, since *d_k_*(*s*) = 0 equates to zero Shannon entropy within the binary derivative hierarchy.

### 3.3. Statistical Independence of the Binary Derivatives

**Theorem** **5.** *If the bits of s follow a Bernoulli process with *P*(s_i_ = 1) = 0.5, then the bits of each derivative dk(s) are statistically uncorrelated and independent, i.e.,:*

E[*s_i_*] = 0.5, Var(*s_i_*) = 0.25, Corr(*s_i_*, *s_i+j_*) = 0, ∀ *j* > 0

**Proof**: This was proven by Davies et al. in 1995 [19]. □

Thus, the binary derivative acts as an entropy-preserving transformation, maintaining the Shannon entropy of the underlying sequence under successive XOR operations.

### 3.4. Independence of Successive Derivatives

**Theorem** **6.** *Because each derivative is composed of statistically independent bits, successive binary derivatives are also independent random variables, preserving the entropy structure across successive levels of differentiation.*

**Proof**: This was again proven by Davies et al. in 1995 [19]. □

This property implies that differentiation does not introduce correlation; rather, it disperses structure—much like a whitening transformation in information theory. Consequently, the binary derivative provides a stable computational framework for analyzing the entropy of binary representations of natural numbers.

### 3.5. Binary Integrands, Complements and Derivatives

**Theorem** **7.** *Every binary number is the binary derivative of exactly two binary numbers of one bit greater length.*

**Theorem** **8.** *The two binary integrands* [20] *are one’s complements of each other.*

**Proof**. By definition, the XOR operator and its iterative extension to strings of arbitrary length *n* yield the same derivative from a string and its one’s complement. Hence, each binary derivative of length 2*^n^* corresponds to a binary number and its one’s complement of length 2*^n^*^+1^. Conversely, each pair of complementary binary integrands of length 2*^n^*^+1^ corresponds to a single binary derivative of length 2*^n^*. Since there are only 2*^n^* binary derivatives of length *n*, the mapping of 2*^n^*^+1^ binary integrands to 2*^n^* binary derivatives must be exactly and uniquely 2:1; otherwise, Theorems 7 and 8 would be contradicted. □

**Corollary** **1.** *Every prime number > 2 expressed in binary has two binary integrands, one bit longer and a single binary derivative one bit shorter.*

**Corollary** **2.** *Following also from Theorems 5 and 6, the primality of a binary number is independent of the primality of its single binary derivative and its two binary integrands.*

**Corollary** **3.** *Every prime expressed in binary has a one’s complement composite twin.*

### 3.6. Termination of the Derivative Chain

**Theorem** **9.** *If* *d_k_*(*s*) = 111…1, *then* *d_k+_*_1_(*s*) = 0.

**Theorem** **10.** *If* *d_k_*(*s*) = 0, *d_k+j_*(*s*) = 0 * for all* 0 < *j* < *n* − *k* − 1.

**Proof**. The proof of Theorems 9 and 10 follows directly from the defined properties of the XOR operation and completes the closure of the derivative sequence. □

**Corollary** **4.** *Due to Theorems 4, 9 and 10, the final binary derivative of every periodic number is zero.*

### 3.7. The Primes Are Equidistributed Across the Binary Derivatives

**Theorem** **11.** *The primes are asymptotically equidistributed across all levels of the binary derivative hierarchy. As n → ∞, the set *P*_n_ = {s ∈ ℕ: s is prime, |s| = n} is uniformly distributed over all possible derivative states d_k_(s).*

**Proof**. By Corollary 2, the primality of a binary number is independent of the primality of both its single binary derivative and its two binary integrands. Crucially, once the first derivative *d*_1_(*s*) of a prime *s* is obtained, all connection with its origin is lost—*d*_1_(*s*) provides no information about whether *s* was prime or composite.

From Theorem 5, the bits of each derivative *d_k_*(*s*) are statistically independent with E[*s_i_*] = 0.5 and Corr(*s_i_*, *s_i+j_*) = 0 for all *j* > 0. By Theorem 6, successive binary derivatives are independent random variables, with XOR-based differentiation acting as a whitening transformation that disperses structure. From Theorems 7 and 8, every binary number is the derivative of exactly two binary numbers (one’s complements), creating a precise 2:1 mapping.

If primes were not equidistributed across derivative states, some derivative pattern of *d_k_* would preferentially correspond to more than one prime. However, the derivative operation destroys all information about primality while preserving statistical independence through a symmetric, entropy-preserving mapping. Therefore, as *n* → ∞, any deviation from uniform distribution vanishes, and primes become equidistributed across all 2*^n−k^* possible states at each derivative level *k*. □

**Corollary** **5.** *The primes are equidistributed across the two states *{0, 1}* of the final binary derivative d_n−*1*_(s).*

## 4. The Entropic Probability of Primality

### 4.1. Definition of the Entropic Probability of Primality

A binary number may not be prime if any of its derivatives *d_k_*(*s*) equals zero (Theorems 1, 2 and 3). The entropic probability of primality therefore measures the extent to which a number avoids zero-entropy or fully periodic states. The final binary derivative is assigned the highest weight due to Theorem 4. The greatest weight is purposefully assigned to the final derivative as periodicity emerges gradually through the derivatives, with the earlier derivatives capturing shorter periodicity (e.g., 11111111) and the later derivatives capturing more complex, longer periodicity, which takes numerous derivative steps, up to and including the final derivative, to evolve to a zero-entropy state. Let

$$z_{k}=\left\{\begin{array}{c} 0,\;if\;H(d_{k}(s))=0\;(i.e.,\;d_{k}(s)=0)\\ 1,\mathrm{otherwise} \end{array}\right.$$

Then we define

(9) $$p\left(s^{\prime }\right)=\sum_{k=0}^{n-1}\left(z_{k}(s)/2^{\left(n-k\right)}\right)+1/2^{n}$$

where the final term corrects for the special case of the Mersenne numbers (*s* = 11…1).

This function, in general, satisfies

0 < p(*s*′) ≤ 1,    **μ**(p(*s*′)) = 2/3,    **σ**^2^(p(*s*′)) = 1/8

p(*s*’) can thus be interpreted as a scale-invariant entropic likelihood that a given number is prime, derived solely from the entropic structure of its binary representation and its binary derivatives. p(*s*’) is a hierarchical weighted average of the Shannon zero-entropy states of the binary derivatives of *s*.

### 4.2. Connection to the Prime Number Theorem

Because p(*s*′) is independent of numerical magnitude and derived only from structural entropy, it complements the analytic form of the Prime Number Theorem (PNT). For numbers of length 2*^k^* bits:

P (*s* is prime) ≈ p(*s*’) · Li(2*^k^*) · 3/2*^k^*^+1^ (10)

and, exactly:

P (*s* is prime) = p(*s*’) · π(2*^k^*) · 3/2*^k^*^+1^ (11)

This formulation integrates entropic structure (via p(*s*′)) with analytic density (via the PNT), providing an entropic, unconditional and computable probability of primality.

### 4.3. Entropic Interpretation

The metric p(*s*′) quantifies the proportion of derivative states that retain non-zero entropy. Primes correspond to high-entropy regions in the derivative space D(*s*), while composites occupy low-entropy basins associated with periodicity. The independence of p(*s*′) from numerical magnitude implies that primality is an intrinsic property of entropy distribution as well as numerical size.

### 4.4. Bounded Stochastic Variance of the Prime Distribution in p(s′) Space

**Theorem** **12.** *Let *P*_n_(p) denote the set of primes of length n with entropic probability *p*(s′) = p. Then the variance of prime density across *p*(s′) partitions is bounded:*

Var[|P*_n_*(*p*)|/|N*_n_*(*p*)|] = ***O***(1/*n*)

*with residuals following a binormal distribution constrained by the Central Limit Theorem.*

**Proof**. Since p(*s*′) = Σ(z*_k_*(s)/2*^n-k^*) where z*_k_*(*s*) ∈ {0,1}, and each z*_k_*(*s*) is an independent Bernoulli variable (Theorems 5 and 6), the Central Limit Theorem applies: p(*s*′) ~ N(2/3, 1/8*n*) as *n* → ∞.

From Theorem 11, primes are equidistributed across derivative space, imposing |P*_n_*(*p*)| = |N*_n_*(*p*)|π(2*^n^*)/2*^n^* + ε_*n*(*p*),_ where ε_*n*(*p*)_ represents stochastic fluctuation with E[ε_*n*(*p*)_] = 0.

The relative variance is Var(|P*_n_*(*p*)|/|N*_n_*(*p*)|) = π(2*^n^*)/2*^n^*·(1 − π(2*^n^*)/2*^n^*)/|N*_n_*(*p*)|. Since |N*_n_*(*p*)| is proportional to 2*^n^* and π(2*^n^*)~2*^n^*/*n* by the Prime Number Theorem, this yields Var(π(*n*)) = ***O***(1/*n*).

The partition p(*s*′) = 1.0 contains exactly half of all numbers. By Corollary 5, primes are equidistributed across the final derivative state, creating independent normal distributions in the upper and lower partitions—a binormal distribution. □

**Corollary** **6.** *Prime distribution exhibits bounded stochastic variance rather than true randomness, with deviations that are asymptotically normal, bounded in magnitude, balanced across binary partitions, and diminishing relative to total prime count.*

**Corollary** **7.** *The bounded variance *Var* = **O***(1/*n*)* *in* *p(*s′*)* space imposes a stronger constraint on prime density fluctuations than the Riemann Hypothesis bound |*π(*x*)* − *Li(*x*)*| = **O***(*√x *log* x*)*. For x = *2*^n^, the entropic variance bound **O***(1*/x*)* = **O***(1/log* x*)* suggests actual deviations are substantially smaller than the von Koch* [21] *bound suggests. This reveals that equidistribution across derivative space forces prime density to track *Li(*x*)* with tighter regulation than analytic continuation alone predicts.*

Interpretation: Theorems 11 and 12 establish that apparent prime “randomness” is actually regulated stochastic behavior, constrained by equidistribution across derivative space, statistical independence, and the Central Limit Theorem.

## 5. Empirical Results

### 5.1. Numerical Evaluation of p(s′)

The metric p(*s*′) was computed for all integers *s* < 65,536 using the algorithmic procedure defined in Equation (11). The calculations were implemented in Microsoft Excel. All worksheets, graphics and tables, together with further supporting materials, are available in the Supplementary Materials. For illustration, we firstly examined a small domain (*s* < 256) to permit direct visual inspection of binary patterns and periodicities. We illustrate the algorithm in Table 1.

Each number < 256 was expressed in binary form, its successive derivatives were computed by XOR differentiation, and the fraction of derivatives with non-zero entropy was accumulated according to Equation (11). The resulting p(*s*′) values were then compared with the actual occurrence of primes in the same intervals and with the predictions of both π(*x*) and Li(*x*).

### 5.2. Primes Below 256

**Figure 1:**
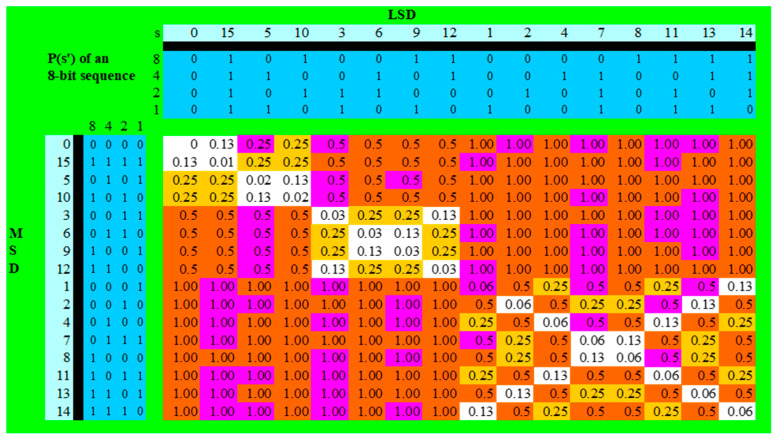
p(*s*′) values for *s* < 256.

For the 8-bit domain (*s* < 256), 54 numbers are prime. Figure 1 below lists the calculated p(*s*′) values, and Table 2 lists the corresponding observed prime counts.

A strong linear relationship was obtained between expected and actual primes, with

*R*^2^ = 0.95   and   *ρ*_Spearman_ = 0.94.

Figure 1 shows the distribution of primes within the p(*s*′) framework for *s* < 256.

Figure 1 is structured such that integers with p(*s*′) ≥ 0.5 are coloured red and integers with p(*s*′) ≥ 0.25 are coloured yellow. Integers with p(*s*′) < 0.25 are white The horizontal and vertical borders give the decimal (white) and binary (blue) values of the Higher (MSD) and lower (LSD) Significant digits. Primes (shaded purple) cluster most preferentially within the region p(*s*′) = 1.0, corresponding to maximal entropy across all derivative levels. In contrast, numbers with p(*s*′) < 0.5 show a marked deficit of primes, consistent with the hypothesis that low-entropy (periodic) binary structures are more likely to be composite.

### 5.3. Extension to s < 65,536

The procedure was repeated for 16-bit numbers. Table 3, Table 4 and Table 5 summarize actual and expected prime counts across discrete p(*s*′) intervals for each power-of-two boundary (2^4^ to 2^16^).

These observed prime distributions display three notable features:

#### 5.3.1. Scale Invariance

The correlation between predicted and observed prime frequencies remains stable (*R*^2^ > 0.93) across all magnitudes, confirming the scale-independent nature of p(*s*′).

#### 5.3.2. Balanced Stochastic Asymmetry

Deviations of actual primes from expectation are evenly but asymmetrically distributed above and below p(*s*′) ≥ 0.5. This indicates bounded stochastic behavior rather than truly random variation.

#### 5.3.3. Diminishing Imbalance

A small stochastic imbalance in the number of primes between the upper and lower binary halves (partitioned by the final derivative value p(*s*′) = 1.0) is clearly observed for small *s* but proportionally diminishes as *s* → ∞ as it must do due to Theorem 11.

### 5.4. Large-Scale Sampling (s < 2^32^)

Ten samples of 2000 randomized 32-bit integers were evaluated to assess the persistence of the predicted and observed trends in p(*s*′). The empirical distribution of p(*s*′) values among the sampled primes was almost identical to p(*s*′) and PNT theoretical expectations. Near-zero residuals between predicted and actual primes in the upper and lower halves of the p(*s*′) distribution were observed.

These findings demonstrate that p(*s*′) functions as a statistically consistent estimator of prime probability across multiple scales.

### 5.5. Prime Density for p(s′)

**Figure 2:**
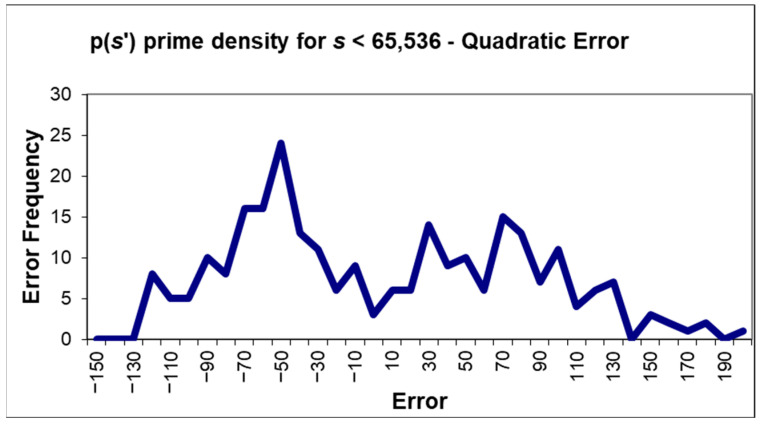
Error *e* between p(s’) prime density and a quadratic curve fit.

Prime density for p(*s*′) for *s* < 65,536 was again calculated [22]. A quadratic curve was fitted to the obtained prime density, and the difference between the two was computed. The distribution of the difference *e* was plotted in Figure 2 and shown to be small and binormal (**μ**(*e*) ≈ 0 and **σ**(*e*) ≈ 56). This was as expected, as the value of p(*s*′) is 1.0 for exactly half of the sample.

## 6. Discussion

### 6.1. Entropy, Periodicity and Primality

Our results reinforce the conceptual view that primes are high-entropy entities within the binary domain. Periodic or low-entropy patterns correspond to compositeness, while the random-like, high-entropy patterns are characteristic of primes. The binary derivative provides a natural operational link between these domains: its XOR-based differentiation process preserves Shannon entropy and reveals any periodic structure through the eventual emergence of zero-entropy derivatives.

From an information-theoretic perspective, composite numbers can be viewed as compressible sequences—they exhibit internal redundancy through repetition—whereas almost all primes behave as incompressible sequences exhibiting continuing maximal disorder under binary differentiation.

### 6.2. Entropic Structure of Number Space

We can consequently divide the number space into differing entropic sets and establish clear relationships between them. We illustrate this in Table 6 below.

### 6.3. Statistical Structure of Number Space

The empirical data indicate that the apparent randomness of primes is statistically constrained. Deviations from analytic expectations in p(*s*′) based prime density analysis are binormal, small, and bounded by the central limit theorem, implying a regulated stochastic process rather than pure randomness. The decreasing asymmetric prime imbalance across binary partitions (which has all but disappeared by 2^32^) further supports the hypothesis that prime variance is asymptotically zero, consistent with the Riemann Hypothesis’ analytic predictions and Theorem 11.

### 6.4. Computational and Cryptographic Implications

Because p(*s*′) can be evaluated directly from a number’s binary representation, it provides a computationally efficient heuristic for testing primality likelihood. While not a deterministic test, it correlates strongly with exact primality and could be combined with conventional probabilistic algorithms (e.g., Rabin–Miller [23] or AKS [24]) to optimize candidate selection in large-scale prime generation.

The entropic interpretation also links number theory with cryptographic entropy assessment, suggesting possible further applications in randomness generation and testing, key validation, and entropy-driven encryption schemes.

### 6.5. Comparison Between p(s′) and BiEntropy

The entropic probability metric p(*s*′) exhibits an almost perfect correlation with the previously defined BiEntropy function (*R*^2^ = 0.9992 for *s* < 256), confirming that both measures capture the same underlying structural information within binary representations. While BiEntropy provides a smooth, almost continuous measure of disorder, p(*s*′) offers a discrete and computationally more efficient formulation. Crucially, p(*s*′) incorporates the final binary derivative, which BiEntropy necessarily omits, thereby revealing the small stochastic asymmetry in the distribution of primes across their terminal derivative states. This capability makes p(*s*′) not only a more precise estimator of primality but also a clearer and simpler analytical expression of the entropic structure of the natural numbers, with Theorems 5–12 easier to argue.

The high correlation between p(*s*′) and BiEntropy demonstrates conceptual continuity with prior work, yet p(*s*′) extends the framework by providing an exact analytic expression rooted in Shannon’s entropy and binary periodicity. Whereas BiEntropy evaluates disorder across derivative layers, p(*s*′) translates that same structure into an exact probabilistic measure with closed-form parameters (**μ**, **σ^2^**). This unification of entropy and periodicity establishes a quantitative bridge between information theory and analytic number theory. The simpler definition of p(*s*′) facilitated the development of the theoretical framework.

### 6.6. Comparison with Rabin–Miller and AKS Primality Tests

The metric p(*s*′) is conceptually distinct from traditional primality tests such as Miller–Rabin [23] and AKS [24]. Whereas the Miller–Rabin test evaluates compositeness probabilistically through modular exponentiation and AKS verifies primality deterministically via polynomial congruences, p(*s*′) is entirely analytic and derived from the intrinsic periodic structure of binary sequences. It requires neither random witnesses nor modular arithmetic, instead estimating the likelihood of primality through the entropy of a number’s binary derivatives. The computation of p(*s*′) is scale-invariant and of complexity ***O***(log_2_(*s*)), making it well-suited for large-scale numerical analysis. Although not a deterministic test, p(*s*′) provides a structural probability of primality that complements, rather than replaces, conventional algorithms such as Rabin–Miller and AKS in computational number theory.

### 6.7. Twin, Fermat, and Mersenne Primes

Application of p(*s*′) to special prime classes demonstrates its consistency with established prime distributions. Under the equidistribution of primes across binary derivatives (Theorem 11), the expected and actual frequencies of twin primes before and after an arbitrary threshold *z* (which might correspond to the last twin prime) must remain in agreement, thereby supporting the twin primes conjecture within the entropic framework. Similarly, Fermat and Mersenne primes arise naturally at densities consistent with p(*s*′) and the Prime Number Theorem, which we demonstrated in the appendices of our prior work. The entropic formulation thus accommodates these prime families seamlessly, reinforcing the generality of p(*s*′) as a unifying descriptor of primality.

### 6.8. The Riemann Hypothesis and Skewes’ Number

The analysis of p(*s*′) provides a complementary entropic interpretation of the Riemann Hypothesis (RH) and related prime-distribution phenomena. The variance of the difference π(*s*) − Li(*s*) is shown to diminish asymptotically as *s* → ∞, implying a tighter convergence than the classical von Koch equivalence π(*s*) = Li(*s*) + O(√*s* log *s*) ⇔ RH. This behavior arises from the stochastic fluctuations in prime density across p(*s*′) partitions, consistent with Littlewood’s theorem [25] that the sign of π(*s*) − Li(*s*) changes infinitely often beyond Skewes’ number [26]. Skewes’ number corresponds to the first zero value of the entropic prime excess in the highest p(*s*′) partition. These findings position p(*s*′) as a scale-invariant and computationally tractable construct linking entropy, periodicity, and the statistical regularities underlying the prime number distribution.

### 6.9. Limitations and Future Work

This study has established an entropic framework linking periodicity and primality in the binary domain. Previous work has already extended these findings to the ternary domain, confirming that the relationship between entropy and primality generalizes across numerical bases. Future research may explore higher-order derivative systems and cross-base entropic interactions to identify deeper invariances in integer structure.

Given that p(*s*′) is scale-invariant with known variance, further investigation could refine bounds for the first occurrence where the entropic prime excess in the highest binary partition equals zero, thereby improving estimates and bounds [27] of Skewes’ number.

Although not a deterministic test of primality, p(*s*′) remains a computationally efficient and analytically grounded heuristic for cryptographic key generation, entropy estimation, and prime-density modeling.

Finally, the transparent spreadsheet-based framework in the Supplementary Materials supporting p(*s*′) offers a set of practical pedagogical tools for demonstrating the interplay between entropy, periodicity, and primality in both research and educational [28] contexts.

## 7. Conclusions

This study introduced an entropic probability of primality, p(*s*′), derived from the periodic and differential structure of binary numbers. Theoretical proofs and empirical analyses collectively demonstrate that p(*s*′) is scale-invariant, analytically well-defined, and strongly predictive of primality. Primes emerge as maximal-entropy states within the binary derivative hierarchy space D(*s*), while composites correspond to entropy minima associated with periodicity.

These findings strengthen the bridge between entropy, randomness, and number theory, providing both conceptual and computational mechanisms for further understanding the mathematical and statistical architecture of the primes.

## Figures and Tables

**Table 1:** Calculating p(*s*′).

*s*	*s*_1_	*s*_2_	*s*_3_	*s*_4_	*s*_5_	*s*_6_	*s*_7_	*s*_8_	1’*s*	*n*	*z*	*k*	2^8−k^	*z*/2^8−k^
23	0	0	0	1	0	1	1	1	4	8	1	0	256	0.0039
	0	0	1	1	1	0	0		3	7	1	1	128	0.0078
	0	1	0	0	1	0			2	6	1	2	64	0.0156
	1	1	0	1	1				4	5	1	3	32	0.0313
	0	1	1	0					2	4	1	4	16	0.0625
	1	0	1						2	3	1	5	8	0.1250
	1	1							2	2	1	6	4	0.2500
	0								0	1	0	7	2	0.0000
													**∑**	0.4961
													Mersenne	0.0039
													p(*s*′)	0.5000

**Table 2:** Actual and Expected primes by p(*s*′) for *s* < 256.

Fraction	p(*s*′)	Expected	Actual	PNT (Li(x))
256	0.0078	0.21	0	0.24
128	0.0156	0.42	0	0.47
64	0.0313	0.84	0	0.95
32	0.0625	1.89	1	1.89
16	0.1250	3.38	0	3.78
8	0.2500	6.75	1	7.56
4	0.5000	13.50	14	15.13
2	1.0000	27.00	38	30.26
Total		54.00	54	60.51

**Table 3:** Actual primes by p(*s*’) for various *s* < 65,536.

p(*s*′)	16	32	64	128	256	512	1024	2048	4096	8192	16,384	32,768	65,536
**≤0.001953**													
**0.003906**						1	1	1	1	1	1	1	1
**0.007813**													
**0.015625**							2	3	5	9	11	23	51
**0.031250**					0	2	2	5	8	14	28	53	95
**0.062500**		1	1	1	1	1	3	5	9	16	31	56	102
**0.125000**						3	8	17	30	62	108	211	403
**0.250000**	1	1	1	1	1	4	9	18	26	79	149	287	494
**0.500000**	1	3	5	10	14	28	41	79	144	264	484	958	1712
**1.000000**	4	6	11	19	36	58	106	181	341	583	1088	1923	3684
**∑ =** **π** **(*s*)**	**6**	**11**	**18**	**31**	**54**	**97**	**172**	**309**	**564**	**1028**	**1900**	**3512**	**6542**
**Note: Li(*s*)**	**9**	**14**	**22**	**36**	**61**	**104**	**181**	**321**	**577**	**1048**	**1920**	**3544**	**6584**

**Table 4:** Expected primes (rounded) by p(*s’*) for various *s* < 65,536.

p(*s*′)	16	32	64	128	256	512	1024	2048	4096	8192	16,384	32,768	65,536
**≤0.000244**													1
**0.000488**												1	2
**0.000977**										1	1	2	3
**0.001953**									1	1	2	3	6
**0.003906**								1	1	2	4	7	13
**0.007813**							1	1	2	4	7	14	26
**0.015625**						1	1	2	4	8	15	27	51
**0.031250**					1	2	3	5	9	16	30	55	102
**0.062500**			1	1	2	3	5	10	18	32	59	110	204
**0.125000**		1	1	2	3	6	11	19	35	64	119	220	409
**0.250000**	1	1	2	4	7	12	22	39	71	129	238	439	818
**0.500000**	2	3	5	8	14	24	43	77	141	257	475	878	1636
**1.000000**	3	6	9	16	27	49	86	155	282	514	950	1756	3271
**∑ =** **π** **(*s*)**	**6**	**11**	**18**	**31**	**54**	**97**	**172**	**309**	**564**	**1028**	**1900**	**3512**	**6542**
**Note: Li(*s*)**	**9**	**14**	**22**	**36**	**61**	**104**	**181**	**321**	**577**	**1048**	**1920**	**3544**	**6584**

**Table 5:** Expected minus Actual primes by p(*s*’) for various *s* < 65,536.

p(*s*′)	16	32	64	128	256	512	1024	2048	4096	8192	16,384	32,768	65,536
**≤0.000244**													−1
**0.000488**												−1	−2
**0.000977**										−1	−1	−2	−3
**0.001953**									−1	−1	−2	−3	−6
**0.003906**						1	1			−1	−3	−6	−12
**0.007813**							−1	−1	−2	−4	−7	−14	−26
**0.015625**						−1	1	1	1	1	−4	−4	0
**0.031250**					−1		−1		−1	−2	−2	−2	−7
**0.062500**		1			−1	−2	−2	−5	−9	−16	−28	−54	−102
**0.125000**		−1	−1	−2	−3	−3	−3	−2	−5	−2	−11	−9	−6
**0.250000**			−1	−3	−6	−8	−13	−21	−45	−50	−89	−152	−324
**0.500000**	−1		1	2	1	4	−2	2	3	7	9	80	77
**1.000000**	1	1	2	4	11	10	20	27	59	69	136	167	413
**∑p(*s*** **′** **) < 0.5**	**0**	**−1**	**−2**	**−6**	**−11**	**−13**	**−18**	**−28**	**−62**	**−76**	**−147**	**−247**	**−489**
**∑p(*s*** **′** **) ≥ 0.5**	**1**	**1**	**3**	**6**	**12**	**13**	**18**	**28**	**62**	**76**	**147**	**247**	**490**

**Table 6:** The Entropic Structure of Number Space.

Set	Entropic Interpretation	Observation
*S* (Periodic Numbers)	Zero entropy p(*s*′) ≈ 0	Composite (*)
*S*′ (Non-PeriodicNumbers)	Non-Zero entropy p(*s*′) > 0	Potentially Prime
*P* (Primes)	All Entropies	Prime
*C* (Composites)	All Entropies	Not Prime
*P* ∩ *S*	∅	Empty Set (*)
*S* ∪ *S*′	Union of all Entropies	Natural Numbers
*S* ⊆ *C*	Lower Entropy	Composite
*P* ⊆ *S*′	Higher Entropy	Prime
*X*	High Entropy p(*s*′) = 1	Potentially prime
*Y*	Low Entropy p(*s*′) < 1	Probably composite
*|X| = |Y|*	High and Low Entropy	Equality of set size
*X* ⊆ *S*′	High Entropy	Potentially prime
*S* ⊆ *Y*	Low Entropy	Composite (*)
*|P|*⊆ *X ≈ |P|*⊆ *Y*	High and Low Entropy	Equality of set size

* Exc. Fermat.

## Data Availability

The data presented in this study are openly available in Figshare at [https://figshare.com/articles/dataset/Aperiodicity_of_the_Primes/21187339], accessed on 23 November 2025.

## Supplementary Materials

The following supporting information is available online at https://figshare.com/articles/dataset/Aperiodicity_of_the_Primes/21187339, accessed on 23 November 2025. Aperiodicity_Primality_of_Nat_Num_256.xls: Complete numerical evaluation of the binary derivatives for all integers *s* < 256. Includes cell-by-cell computation of p(*s*′), *z*_k_(*s*). Graphic illustration of the distribution of the primes by p(*s*′). Means, counts and variances of key parameters, including derivatives, primes, twin primes and associated graphics. P_s_prime_64k_calc_v1.45.xls: Complete numerical evaluation of p(*s*′) for all integers < 65,536. Includes cell-by-cell computation of p(*s*′). Graphic illustrations of the distribution of the primes by p(*s*′). Means, counts, variances and regressions of key parameters. Contains comparison with π(s), Li(s) and Prime Number Theorem predictions. Binormal error distribution of p(*s*′). P_S_Prime_32_Bit_Calculator_v1.00.xls: Full 32-bit p(*s*′) calculator including primality test. P_S_Prime_32_Bit_Test_V1.00.xls: Test set generator for 32-bit randomized samples. P_S_Prime_32_bit_Test_Results.xls: Test set results for 20,000 32-bit samples. Appendix_B_v1.01.xls: Illustration of the periodicity, *n*-periodicity and *z*-periodicity of the 8-bit integers. Appendix_C_v1.01.xls: Enumeration of the counts of integers < 65,536 deterministically precluded from primality due to Theorems 1, 2 and 3. Together, these materials permit full reproduction and validation of every result, figure, table and comment reported in the main text. They provide additional datasets and analyses.
